# Investigation of Parameter Effects on Virtual-Spring-Force Algorithm for Wireless-Sensor-Network Applications

**DOI:** 10.3390/s19143082

**Published:** 2019-07-12

**Authors:** Zhiyong Yu, Rongxin Tang, Kai Yuan, Hai Lin, Xin Qian, Xiaohua Deng, Shiyun Liu

**Affiliations:** 1 Institute of Space Science and Technology, Nanchang University, Nanchang 330031, China; 2 Lunar and Planetary Science Laboratory, Macau University of Science and Technology, Macau 999078, China; 3 College of Physical Science and Technology, Central China Normal University, Wuhan 430079, China; 4 One Microsoft Way, Redmond, WA 98052-6399, USA; 5 Usun Microelectronics, Nanchang 330072, China

**Keywords:** wireless sensor network, virtual spring force, pair correlation diversion, parameter analysis

## Abstract

Virtual-force algorithms (VFAs) have been widely studied for accurate node deployment in wireless-sensor-network (WSN) applications. Their main purpose is to achieve the maximum coverage area with the minimum number of sensor nodes in the target area. Recently, we reported a new VFA based on virtual spring force (VFA-SF) and discussed in detail the corresponding efficiency via statistical analysis. The optimized strategy by adding an external central force (VFA-SF-OPT) was presented, which effectively eliminates the coverage hole or twisted structure in the final network distribution. In this paper, the parameter effects on VFA-SF and the VFA-SF-OPT were further investigated: (1) Node velocity dramatically affects the convergence rate of the node-deployment process. (2) A suitable external central force improves equilibrium distance and reduces energy consumption. (3) The effects of VFA-SF and VFA-SF-OPT for different types of obstacles are discussed. Generally, by choosing suitable parameters, both VFA-SF and VFA-SF-OPT can effectively improve node deployment and energy consumption for the whole sensor network. The results give important insight in parameter selection and information fusion in the application of a large-scale WSN.

## 1. Introduction

With the rapid development of wireless sensor networks (WSNs), the accurate deployment of a large number of wireless sensors would become an important issue in the near future, e.g., the application of unmanned aerial vehicles (UAVs) or autonomous underwater vehicles (AUVs) [1,2]. In recent years, many researchers have focused on the problem of position optimization in WSNs since node deployment significantly affects both system lifetime and energy consumption in a two-dimensional (2D) environment. It was proved that a hexagonal structure is the best topology in a 2D network. It provides the maximum coverage area with the minimum number of sensor nodes and minimum energy consumption [3].

The virtual-force algorithm (VFA) is one of the most popular mechanisms for node deployment, in which sensors are moved by the virtual force determined by the relative position of neighbor nodes and the presence of an event [4,5,6]. The VFA presents a force-directed approach to improve network coverage with limited sensor nodes. It has low computation complexity with less computation time and one-time repositioning for the sensor nodes. Heo and Varshney improved the virtual-force algorithm by adding certain constraints to the virtual force function [7]. Based on the theory of virtual potential field and disc packing, Chakrabarty and Zou used attractive and repulsive forces to determine the virtual motion paths and movement rates of the sensor nodes. A probabilistic target-localization algorithm was presented to enhance field coverage [5,8]. Kribi et al. reported a serialized VFA, like the $L_{max}$-serialized VFA and the $D_{th}\_L_{max}$-serialized VFA. It has good coverage, connectivity, and fault-tolerance to increasing the robustness of a robot sensor network [9]. In order to quickly respond to sudden events or disasters, Garetto et al. investigated a distributed relocation method with regular tessellation of the geographical area [6]. Yu et al. introduced a newly algorithm based on Van der Waals force [10], which yielded good uniformity, field coverage, and system convergence.

In general, the fixed coverage area and model parameters are provided as the system inputs to the VFA, which cause a flexible and efficient solution in real WSN applications. However, the node location affects the calculations of node velocities and accelerations. In such a case, location accuracy is important in common VFAs. It is necessary to further discuss the effects of system parameters on the localization of sensor nodes. Tang et al. implemented the algorithm computation and analyzed the details of influence of experimental parameters on Garetto’s algorithm [11]. They also presented a self-consistent method by leveraging the phenomena in which dusty particles can automatically form hexagonal topology. The influence of the computation scale and shielding length on this algorithm were also investigated [12].

Recently, we presented a virtual-force algorithm inspired by spring force (VFA-SF), in which the sensors are moved by the virtual spring force, determined by the relative position of neighboring nodes. After statistically analyzing the stability of the VFA-SF, we adopted an optimization solution (VFA-SF-OPT) where node deployment begins from the center region by adding an external force to the most peripheral nodes of the network. Simulations showed that the VFA-SF-OPT finally yields perfect hexagonal topology [13].

However, in our previous paper, the theoretical exercises were carried out as proof of concept in the VFA-SF and the VFA-SF-OPT. In the simulated scenarios, the entire area was covered. It is still important to extensively discuss how parameters can potentially affect the performance of node deployment. This paper mainly aims at answering three questions: (1) Since location accuracy is related to node velocity in the algorithm process, what is the influence of node velocity on network topology and system convergence? (2) What is the impact of different values of the externally applied force for the optimized algorithm? (3) What is the effect when obstacles are within the area of interest? Based on these questions, we carried out related simulation experiments to evaluate such effects on the performance and accuracy of the proposed algorithms. The results showed that suitable parameters can effectively improve coverage performance for the network, while a high overall coverage ratio is a goal.

The rest of this paper was organized as follows. The related virtual-spring-force algorithm and corresponding optimized method are introduced in Section 2. Section 3 proposes the methodology of performance evaluation. The simulation results of different parameter effects during the deploying process are presented in Section 4. Finally, the conclusion and directions for future work are summarized in Section 5.

## 2. Theory of Virtual-Spring-Force Algorithms

In this section, the theoretical description for the virtual-force algorithm based on spring force and the corresponding optimization method are introduced. All sensors were assumed to be of identical capabilities in sensing, communication, computation, and mobility. Each sensor is capable of moving by itself and detecting its own location with some method (e.g., the Global Positioning System (GPS)). The detailed process of the VFA-SF was presented in our previous paper [13].

### 2.1. Original Virtual-Spring-Force Algorithm

Communication range $R_{c}$ and sensing range $R_{s}$ are the characteristic parameters of each sensor. In a perfect hexagon topology, equilibrium distance $D_{m}$ between two neighbor nodes should be $\sqrt{3}R_{s}$. Therefore, communication range $R_{c}$ is usually larger than $\sqrt{3}R_{s}$.

At the beginning of the deployment process, all *N* nodes in a wireless sensor network were randomly deployed in the target region whose area was *S* and the center was located at (0,0). The location of each sensor $n_{i}$(i=0,1,…,N−1) is given by a vector ${\vec{x}}_{i}\left(t\right)$ (${\vec{x}}_{i}\left(t\right)$ also indicates the position of node *i* at time *t*). Vector ${\vec{d}}_{ij}\left(t\right)$ indicates the distance between node *i* and node *j* at time *t*.

The force on node *i* at time *t* is driven by the Newton motion law:

(1) $$m\frac{d^{2}{\vec{x}}_{i}\left(t\right)}{dt^{2}}={\vec{F}}_{i}\left(t\right)$$

where *m* is the mass of a sensor node, and ${\vec{F}}_{i}\left(t\right)$ is the resultant force. ${\vec{F}}_{i}\left(t\right)$ can be defined as the sum of three components:

(2) $${\vec{F}}_{i}\left(t\right)={\vec{F}}_{i}^{e}\left(t\right)+{\vec{F}}_{i}^{f}\left(t\right)+{\vec{F}}_{i}^{ce}\left(t\right)$$

where ${\vec{F}}_{i}^{e}\left(t\right)$ is the virtual spring force, ${\vec{F}}_{i}^{f}\left(t\right)$ is the damping force, and ${\vec{F}}_{i}^{ce}\left(t\right)$ is the centripetal force.

Generally, spring forces are established between pairs of sensor nodes if one node has no other nodes in the upper and lower 60 degree sectors with another node, and their distance is smaller than $R_{c}$. Here, ${\vec{x}}_{ij}\left(t\right)=\left({\vec{x}}_{j}\left(t\right)-{\vec{x}}_{i}\left(t\right)\right)/d_{ij}\left(t\right)$ is the normalized vector from node *i* to *j*. If the set of possible node *j* acting with virtual forces on node *i* ($d_{ij}\left(t\right)<R_{c})$ is expressed by $\Phi_{i}\left(t\right)$, the corresponding total spring force of node *i* is

(3) $${\vec{F}}_{i}^{e}\left(t\right)=\sum_{j\in \Phi_{i}\left(t\right)}\kappa \left(d_{ij}\left(t\right)-D_{m}\right){\vec{x}}_{ij}\left(t\right)$$

where κ is the spring coefficient.

Damping force ${\vec{F}}_{i}^{f}\left(t\right)$ is defined as

(4) $${\vec{F}}_{i}^{f}\left(t\right)=\left\{\begin{array}{c} -\min\left(\gamma_{0}{\vec{x}}_{0}\left(t\right),\;{\vec{F}}_{i}^{e}\left(t\right)+{\vec{F}}_{i}^{ce}\left(t\right)\right),\;\;\mathrm{when}\;\;d{\vec{x}}_{0}\left(t\right)/dt=0\\ -\gamma d{\vec{x}}_{i}\left(t\right)/dt,\;\;\;\mathrm{otherwise} \end{array}\right..$$

Damping force reduces the elastic potential energy for the whole network and accelerates the convergence of the node deployment. The upper formula is the case when a sensor node approaches a quasiequilibrium state since the node velocity $\vec{v}=d{\vec{x}}_{0}\left(t\right)/dt=0$. In such a case, the damping force equals to $-({\vec{F}}_{i}^{e}\left(t\right)+{\vec{F}}_{i}^{ce}\left(t\right))$ (force balance) or $-(\gamma_{0}{\vec{x}}_{0}\left(t\right))$. Once the damping of the spring oscillator is absent, the oscillator should work in a simple harmonic vibration state; $\omega_{0}$ is the natural vibration frequency, and $\gamma_{0}$ is the natural damping coefficient. The lower formula is the case during the algorithm processing. In such a case, damping force is related to node velocity and γ is the given damping coefficient.

Let $\kappa /m=\omega_{0}^{2}$ and γ/m=2β, where β is the real vibration frequency, related to the damping effect. Usually, damping force strengthens with the increase of β. New quantity ε is defined as $\varepsilon =\gamma /(2\sqrt{\kappa m})$. When ε=1 and $\beta =\omega_{0}$, the sensor network operates in a critical damping condition. Elastic potential energy decreases due to the damping force, and the system quickly converges to an equilibrium state without unnecessary vibration or energy consumption. Under this circumstance, the critical value of parameter $\gamma_{critical}=2\sqrt{\kappa m}$.

Reference [14] has already discussed the influence of damping coefficient γ on the virtual-spring-force algorithm. They tried a fixed γ during all deployment and also varied the γ in different stages of deployment process. The results suggested that each damping condition, with its unique characteristic, could play to its strengths in different deployment stages in real applications. Therefore, in this paper, damping coefficient γ is not discussed. We then followed their work and also used a dynamic damping coefficient. In time steps 0–300, $\gamma =1.08\times \gamma_{cirtical}$, and after 300 time steps $\gamma =\gamma_{cirtical}$.

Centripetal force is defined as

(5) $${\vec{F}}_{i}^{ce}\left(t\right)=-F_{centri}\times {\vec{x}}_{i}\left(t\right),$$

which is only an external auxiliary force acting on each sensor node. With centripetal force, sensors move closer to the region center and always return to the sensor networks. The $F_{centri}$ constant must be much smaller than spring force so that it does not affect the main virtual algorithm. Once a wireless sensor network is deployed to the final hexagon topology, this force is released in the algorithm.

A second-order leapfrog scheme was utilized for the time discretization in Equation (1), as follows:

(6) $$\begin{array}{ccc} {\vec{r}}_{i+1} & = & {\vec{r}}_{i}+{\vec{v}}_{i}dt+{\vec{a}}_{i}{\left(dt\right)}^{2}/2 \end{array}$$

(7) $$\begin{array}{ccc} {\vec{v}}_{i+1} & = & {\vec{v}}_{i}+\left({\vec{a}}_{i}+{\vec{a}}_{i+1}\right)dt/2 \end{array}$$

where $\vec{v}$ and $\vec{a}$ are particle velocity and acceleration at each step, respectively. After obtaining all the position information of neighbors, the sensor node can calculate *r* and *v* using Equations (1) and (2). For completeness, the pseudocode of the leapfrog schema solution for VFA-SF within the wireless sensor network is shown in Algorithm 1. To prevent the description from becoming cumbersome, we omitted details that could either be inferred or implemented using standard techniques.

**Algorithm 1:** Distributed Algorithm for Leapfrog in Virtual Spring Force (VFA-SF) in Wireless Sensor Networks.

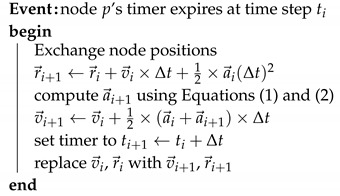



### 2.2. Optimized VFA-SF Strategy

In the original VFA-SF, the self-deployment process calculates spring forces, positions, and movements for all nodes at each time step. It has a 60% possibility with some coverage holes or twisted balance in the final deployment distribution.

The optimized VFA-SF strategy (VFA-SF-OPT) begins node redeployment from the central region since node deployment in central region is more important than that near the edge. At the early stage of deployment, external force $F_{extern}$, whose direction points to the center (0,0), is added to the most peripheral nodes of the wireless sensor network. Other sensor nodes in the external region are gradually involved to the deployment optimization.

External forces should have the following characteristics: (1) always applied to the outermost node of the sensors participating in the deployment algorithm; (2) due to the spring effect, external forces acting on the most marginal node are transmitted to the internal network; (3) similar to centripetal force, external forces should be released when network deployment is to be completed. It promotes the formation of perfect hexagon topology and effectively avoids holes or twisted balance.

The shift from global computation and deployment to centrally preferred deployment was very stable and effective. Further statistical analysis also showed that all wireless sensor networks, regardless of what the initial distribution is, finally yield perfect hexagonal topology. Optimized redeployment can work for nonstandard deployment and yield lower energy consumption than that of the original virtual-spring-force algorithm [13].

## 3. Performance Evaluation of the VFA-SF

In the present study, a novel performance metric based on the pair correlation function within the crystalline structure was introduced to evaluate the topology of the sensor network. Pair correlation function g(r)=(N(r,Δ)S)/(2πrΔN) within the network structure represents the probability of finding two nodes separated by a distance *r*, where *S* is the area of coverage, *N* is the number of sensor nodes contained within *S*, and N(r,Δ) indicates the number of nodes located between r−Δ/2 and r+Δ/2. Therefore, the pair correlation diversion is

(8) $$\delta \left(\Omega ,\Omega_{H}\right)=\frac{\int_{0}^{r_{T}}{\left\|g_{\Omega }\left(r\right)-g_{\Omega_{H}}\left(r\right)\right\|}^{2}dr}{\int_{0}^{r_{T}}{\left\|g_{\Omega_{H}}\left(r\right)\right\|}^{2}dr}\;,$$

where Ω is the network topology to be analyzed, $\Omega_{H}$ is the perfect hexagonal topology, and $r_{T}$ is the bound of the radial distance.

Yu et al. already compared pair correlation function g(r) for network topology resulting from the original VFA-SF with that of a perfect hexagon. The positions of sensor nodes from the VFA-SF were consistent with the required locations from the perfect hexagon [14]. Therefore, pair correlation diversion (PCD) provides an objective metric for evaluating the virtual-force algorithms. It quantitatively analyzes the proximity from any network topology to a perfect hexagon configuration [11,12]. This metric indicates the uniformity for the sensor network. Moreover, it can be used to characterize the convergence rate of any virtual-force algorithm. The slope of the pair-correlation-function curve, as a function of the simulation steps, indicates how fast the virtual-force algorithm converges.

For a wireless sensor network, when the value of $\delta (\Omega ,\Omega_{H})$ is small, the network coverage topology approximates a hexagonal structure. Generally, a perfect deployment solution has a PCD value of 0. In addition, the convergence rate can be obtained according to the decrease of the PCD curve. When a network reaches its steady state, the PCD value should also become stable.

## 4. Parameter Effects on VFA-SF and VFA-SF-OPT

As for the initial simulation parameters, the number of sensor nodes N=500 was chosen in the target region. The mass of each sensor is normalized to m=1 without considering the hardware design and the mechanical parts of the sensor node. Sensing range is also normalized to $R_{s}=1$. In such a case, the final coverage of the whole wireless sensor network relies on the real sensing range of the given sensors. Communication range $R_{c}$ is also normalized based on the $R_{s}$. In order to calculate spring forces from the closest neighbor nodes only, we set $R_{c}=3R_{s}=3$ as shielding length. We then set spring coefficient κ=15 and $F_{centri}=0.005$, which correspond to the equilibrium distance $D_{m}=\sqrt{3}$, $\gamma =\sqrt{4\kappa m}=7.75$.

Moreover, based on these parameters, the theoretical external force, which always points to the center, is 2.873 (see Figure 6 in the reference [13]). This force can break the distribution balance during the deployment process. In our previous paper, external force $F_{extern}=3.5$ was used to successfully eliminate coverage holes without affecting network formation.

The detailed information of simulation parameters and algorithm process can be found in our recent paper [13]. In this section, in order to further improve our VFA-SF and VFA-SF-OPT algorithms for the real field test in WSN applications, the parameter effects on these two algorithms are carefully discussed. Table 1 gives the details of the initial parameters in the following experiments.

### 4.1. Effect of Node Equilibrium Distance

In most WSN applications, sensor communication and the corresponding routing protocol affect system performance, so they are very important topics in theoretical modelling and field tests. In this paper, we do not consider any communication and mechanical parts since our VFA-SF and VFA-SF-OPT are a concept of a node-deployment solution. If a designer wants to reduce the errors from the node position or neighbor location not updating instantaneously, it is better to require each sensor node to wait for a while to receive the updating information from neighbor nodes after it approaches the estimated position. In such a case, in order to reduce the influence of the location error, it can be solved by controlling the node equilibrium distance.

**Figure 1:**
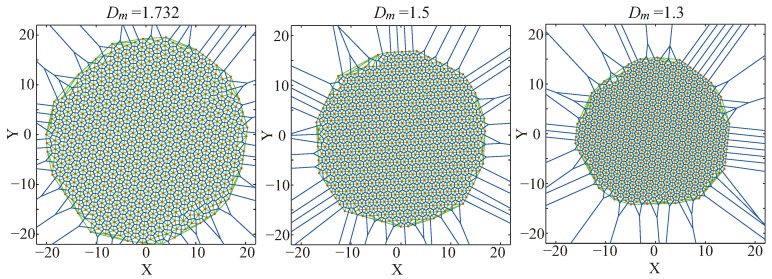
Final node distributions by the VFA-SF for indicated node equilibrium distance $D_{m}=1.732$, 1.5, and 1.3, respectively. Corresponding coverage area by these 500 nodes is 1299, 974.1 and 732, respectively.

Figure 1 shows the final node distributions by the VFA-SF for the indicated node equilibrium distance $D_{m}=1.732$ (theoretical value), 1.5, and 1.3, respectively. The corresponding coverage area by these 500 nodes is 1299, 974.1, and 732 from the area of total hexagon cells. PCD values are 0.03, 0.06, and 0.09. It is obvious that a small $D_{m}$ causes system redundancy and reduces the whole coverage area. Most importantly, a small $D_{m}$ compresses node distance so that the final network may become worse. In such a case, for a fixed target area, a small $D_{m}$ can dramatically avoid location errors but increase the number of sensor nodes and amount of system consumption.

### 4.2. Effect of Node Velocity

Node velocity is calculated at every time step, while node locations and spring forces are determined from the VFA-SF or VFA-SF-OPT. A series of theoretical exercises were carried out to prove the validation of two algorithms, but accuracy is very important to optimal coverage. How node velocity can potentially affect deployment performance needs to be further discussed. Here, the experiments and statistical analysis were implemented to show the effect of node velocity on the VFA-SF (external central force $F_{extern}=0$).

Sensor networks used to have coverage holes or twisted structures during the deployment process, especially at the early stage. The distance between two nodes in such a region is possibly much larger than the average distance. The corresponding node velocity, determined by spring force, can be large. Therefore, maximum node velocity has to be limited in the virtual-spring-force algorithms in case the sensor node has an unreasonable speed. In the simulations, maximum node velocity $v_{max}$ is given as a constant. If the determined node velocity is larger than the fixed value of $v_{max}$ at one time step, it would be reset to be $v_{max}$.

**Figure 2:**
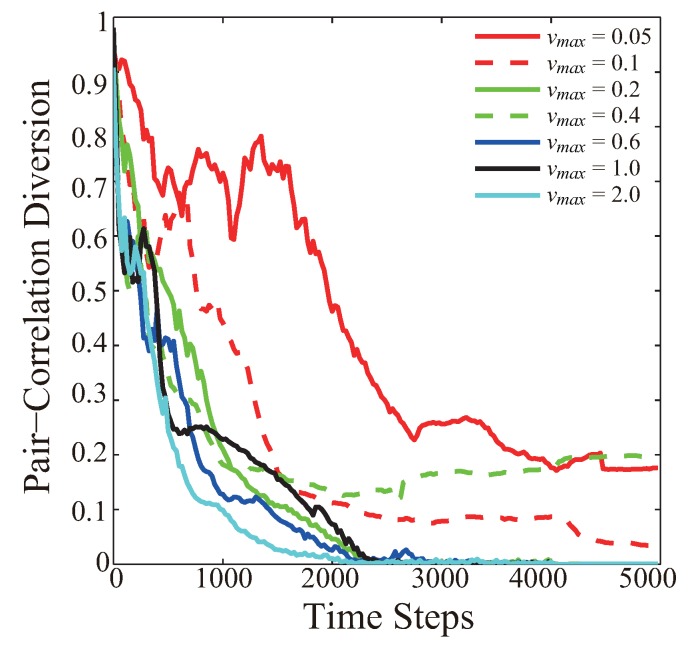
Pair correlation diversion as a time-step function for the indicated maximum node velocity $v_{max}$ = 0.05, 0.1, 0.2, 0.4, 0.6, 1.0, and 2.0, respectively.

Figure 2 presents the pair correlation diversion as a time-step function for indicated maximum node velocity $v_{max}=0.05,0.1,0.2,0.4,0.6,1.0,$ and 2.0, respectively. The comparison is based on the identical initial node distribution. When $v_{max}$ is very small, like $v_{max}$ = 0.05, the PCD curve fluctuates at the early stage of the VFA-SF process. It decreases to 0.3 at the 2500th time step, and finally stays around 0.2. The reason is that a large number of sensor nodes can easily reach the limitation of a small $v_{max}$, which significantly responds to a short moving distance for each sensor at any time step. In such a case, sensor nodes have to spend more time and consume more energy in the deployment process to form quasihexagonal topology. When $0.1\leq v_{max}\leq 0.4$, the PCD curves can finally stay around 0–0.2, which means that a medium $v_{max}$ helps improve the effect of node deployment in some extent. However, this improvement is still unstable. When $v_{max}\geq 0.6$, the final WSN deployment successfully forms a perfect hexagonal topology (PCD value goes to 0). Most importantly, it should be noted that the PCD curves converge quicker as the $v_{max}$ limit increases.

**Figure 3:**
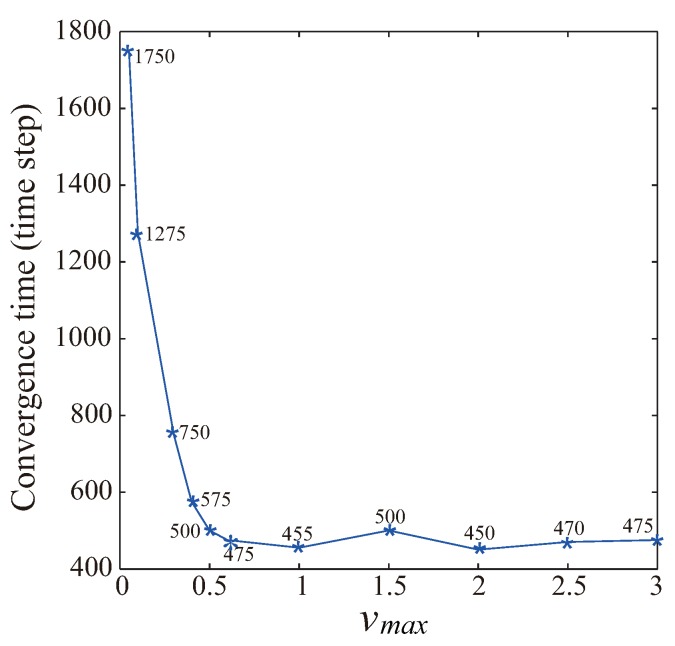
Variation of convergence time as a function of $v_{max}$ for the case of PCD =0.4.

Figure 3 shows the variation of convergence time as a function of $v_{max}$ for the case of PCD = 0.4. Based on statistical analysis, 0.4 is a typical value for the PCD performance, in which the network nearly forms a quasihexagonal topology but still has holes or twisted structures in some small regions. After that, designers can use the VFA-SF-OPT to further optimize deployment. Therefore, 0.4 is a good indication to evaluate the variation of convergence time for different values of $v_{max}$. When $v_{max}<0.5$, convergence time dramatically decreases from 1750 to 500 time steps as $v_{max}$ increases. When $v_{max}>0.5$, only a few sensor nodes exceed the $v_{max}$ limit. It has ignorable impact on the convergence rate of the whole deployment process.

**Figure 4:**
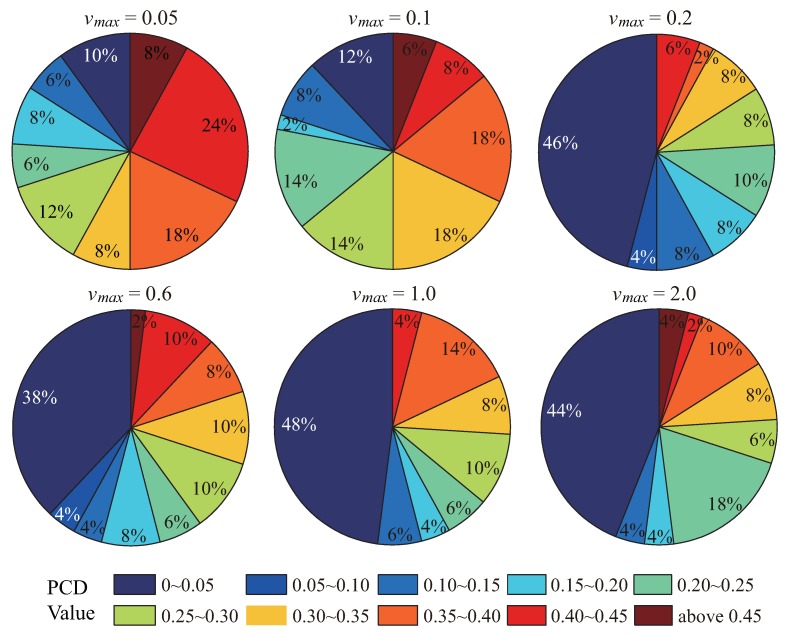
Corresponding possibilities of the final PCD value for different values of $v_{max}$.

Moreover, statistical results of PCD values evaluated from the final network topology by the VFA-SF with $v_{max}=0.05,0.1,0.2,0.6,1.0,$ and 2.0 are listed in Table 2. Fifty independent cases with different initial node distributions were randomly chosen for statistical analysis. Figure 4 also presents pie charts with percentages of the final PCD value for different values of $v_{max}$. When $v_{max}<=0.1$, there was only 10–12% possibility with final PCD values of 0–0.05, which corresponds to a perfect hexagonal topology. The PCD value was calculated in the most central area of the target region, but was only lacking at the edge of the sensor network to some extent. This means that a smaller $v_{max}$ causes worse node deployment in WSN applications, which is consistent with Figure 1. When $0.2<v_{max}<2.0$, the possibility of the PCD value of 0–0.1 is stable at 42–50% (the white values in the pie charts of Figure 3), which corresponds to the perfect hexagonal network. A larger $v_{max}$ helps to improve the final network deployment but does not increase the possibility of perfect hexagonal topology.

### 4.3. Effect of External Central Force

**Figure 5:**
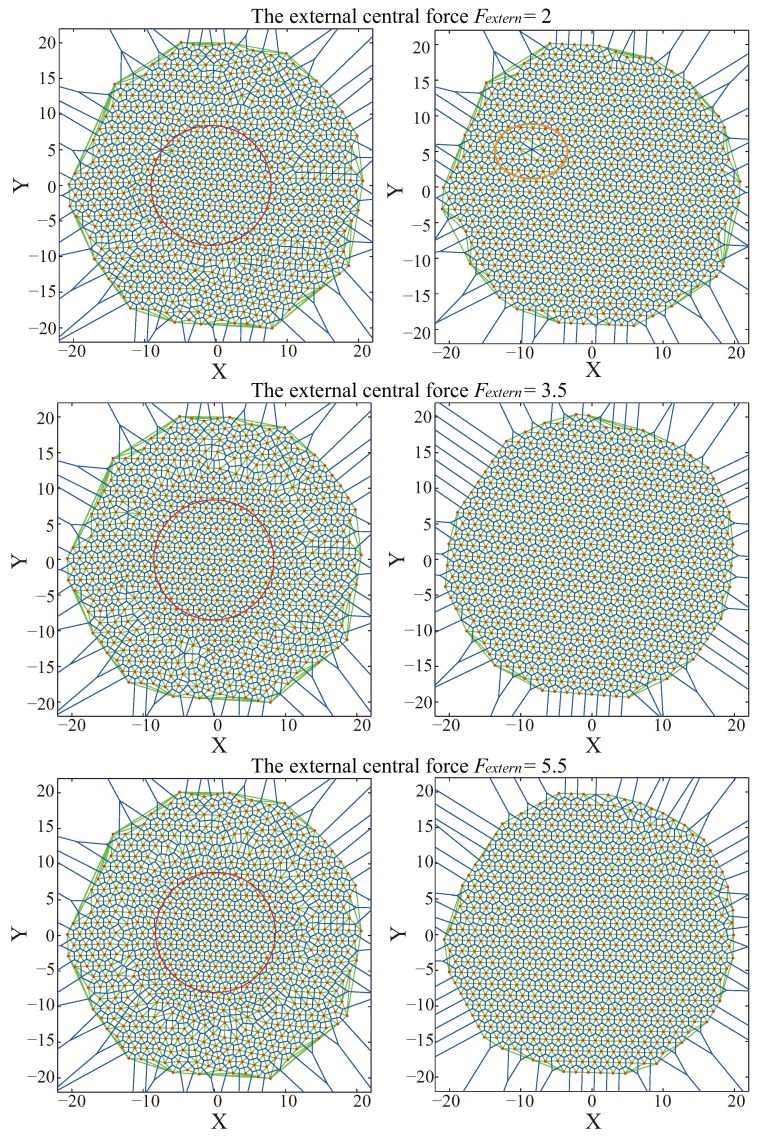
Typical simulation results of node deployment by the VFA-SF-OPT for the indicated external central force $F_{extern}=$ 2.0, 3.5 and 5.5, respectively. Left panels, deployment at 600th time step. Right panels, final network topology at 5000th time step.

**Figure 6:**
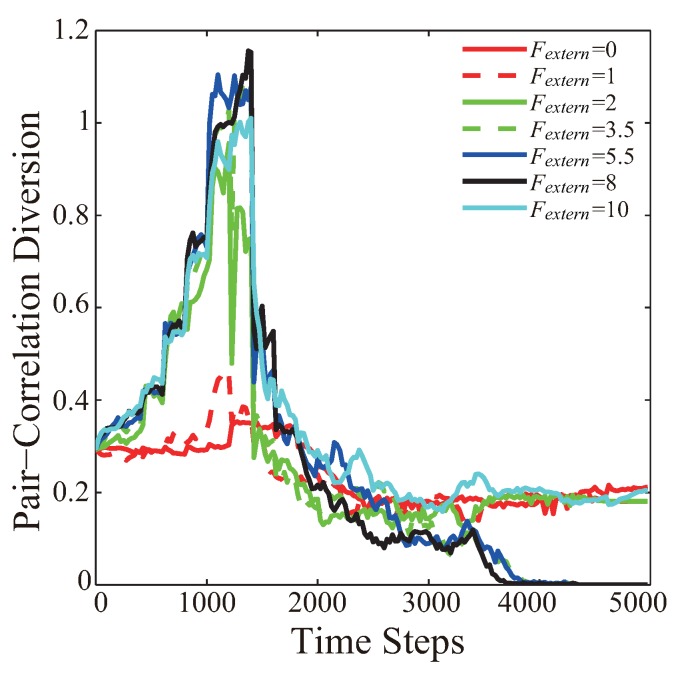
Variation of the PCD curves from VFA-SF-OPT optimization for indicated external central force $F_{extern}$ = 0, 1, 2, 3.5, 5.5, 8 and 10, which correspond to the cases in Table 2.

The calculation of the external central force in Figure 6 of our previous paper [13] was a theoretical indication. On the other hand, network-deployment experiments may not require a very precise value. Usually, designers can choose a suitable value, slightly greater than the optimal value. However, the choice of external central force does have important impact on VFA-SF-OPT optimizations. Typical simulations for three different values of external central force are shown in Figure 5.

The top panels show the deployment topology for $F_{extern}=2$. The left panels are the network topologies in the middle of the deployment process (VFA-SF-OPT) at the 600th time step. The right panels are the final networks after VFA-SF-OPT optimization. Red circles in the left panels indicate the region involved in the calculation of the PCD curve and the equilibrium distance at the 600th and 5000th time step to evaluate the algorithm performance. It is very clear that, if the external virtual force is small, it is still hard to eliminate the coverage hole in the final node distribution (see the orange ellipse in the top-right panel), as well as the twisted structure. The reason is that small external force may not contribute enough to the total force, which drives the sensor to the optimal position within the fixed calculation time. The middle panels illustrate the results corresponding to $F_{extern}=3.5$ that were presented in a previous work [13]. Bottom panels show the results corresponding to $F_{extern}=5.5$. Larger external force can usually yield good hexagonal topology in a large-scale WSN.

However, it should be noted that the gap between the deployed nodes by the VFA-SF-OPT and those nodes outside of the calculation region increases when the external central force becomes larger. The reason is that the VFA-SF-OPT only calculates the positions and the total virtual force to drive the node movements for the sensors involved in the optimization process. Sensor nodes within the optimization region follow the action of the external force while the rest do not temporally move. Centrally preferred deployment can effectively reduce energy consumption for sensors excluded from the VFA-SF-OPT.

Furthermore, average equilibrium distance and average moving distance within the region of radius = 10 for the indicated values of the external central force from the VFA-SF-OPT are listed in Table 3. If $F_{extern}=0$, equilibrium distance $D_{m}\approx \sqrt{3}$, which is consistent with theoretical analysis of the original VFA-SF. When external central force increases, the average equilibrium distance between two neighbor nodes becomes smaller. It means that a larger external central force causes the compression of the central area during the process of optimization deployment. It is consistent with the gap variation in Figure 3. In such a case, those nodes that are newly involved in the VFA-SF-OPT process have to move for a longer distance, which consequently leads to more energy consumption. That is also consistent with Column 3 of Table 3. The final topology may also not be regular hexagonal topology, and it is difficult to recover after releasing this external force in the optimization algorithm.

Generally, extremely large or small value of external central force positively or negatively affects the energy consumption of the sensor nodes, and impacts system performance. It can be determined based on the requirements of environment scenarios or location accuracy for some real WSN applications in a large target area.

Figure 6 presents the variation of PCD curves from the VFA-SF-OPT optimization for indicated external central force $F_{extern}=$0, 1, 2, 3.5, 5.5, 8 and 10, which correspond to the cases in Table 3. When external central force is larger, the PCD value dramatically increases at the early stage of the VFA-SF-OPT. The reason is that the nonhexagonal structure in the central area significantly affects PCD calculation. Larger external central force compresses the central area and causes a broader gap between the nodes involved in the VFA-SF-OPT and those absent from the VFA-SF-OPT. The PCD value reaches its maxima around the 1400th time step.

It should be noted that, once the external force is smaller than the theoretical value ($F_{extern}$ = 2.873 in [13]), like $F_{extern}$ = 0, 1, 2, the PCD curve cannot be reduced to be 0, i.e., a perfect hexagonal structure cannot be achieved. The reason is that a small $F_{extern}$ makes less-significant contribution to the total virtual force and finally yields the survival of the coverage holes. An extremely large external force, like $F_{extern}$ = 10, excessively compresses the central region and breaks the balance of the hexagonal structure. In addition, sensor nodes need to move for a longer distance in such a case.

In addition, two more values of the external central force have been tested to further check the influence of this effect on the VFA-SF-OPT. The corresponding average equilibrium distances and average moving distances are shown in Table 4. It is still obvious that, when $v_{max}=0.3$ and 1.0, the variations of the average node-equilibrium distance and the average moving distance are the same as those of $v_{max}=0.6$. However, in the VFA-SF-OPT ($F_{extern}\neq 0$), all moving distances from $v_{max}=0.6$ are smaller than those from the other two cases. It means that an appropriate value of $F_{extern}$ has less energy consumption in the optimization process.

### 4.4. Effect of Different Obstacles

In order to present our statistical analysis for the VFA-SF and the optimized method VFA-SF-OPT for the first time, optimal network deployment was only considered to cover the whole target area without obstacles. However, in physical WSN applications, obstacles exist. It is very important to discuss the effect based on the different obstacle types. Experiments with some typical obstacles are shown in the figures, implemented by using both the VFA-SF and the VFA-SF-OPT.

**Figure 7:**
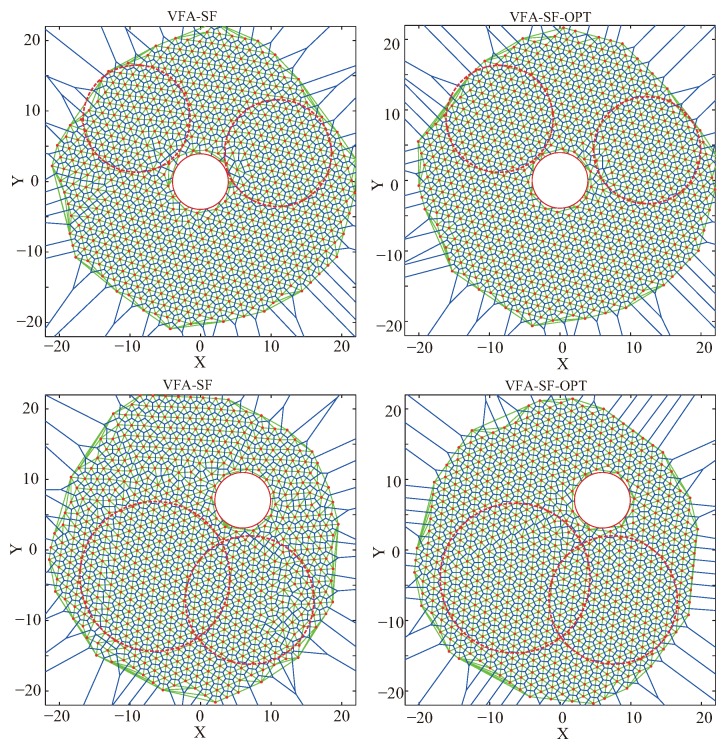
Final node deployments from (**left**) the VFA-SF and (**right**) the VFA-SF-OPT for the circle obstacle cases: (**top**) when circle obstacle is at the center of the target area; (**bottom**) when the circle obstacle is off-center. Red dashed circles, region used to calculate the PCD values.

Figure 7 presents the final node deployments from the VFA-SF (left panels) and the VFA-SF-OPT (right panels) with a circle obstacle. Top panels are the cases when the obstacle was at the center, and bottom panels are cases when the obstacle was off-center. It is clear that the VFA-SF-OPT had a better deployment result than the VFA-SF referring to network topology. The right panels had more perfect hexagonal cells than the left panels.

However, it should be noted that network structures with the off-center obstacle were worse than the cases with the obstacle at the center. The reason is that both the VFA-SF and the VFA-SF-OPT calculate the total virtual force and the corresponding physical velocity and acceleration based on the hexagonal network assumption. The six neighbor nodes were distributed around a given node like a circle. The external central force added in the VFA-SF-OPT pointed to the center of the target region, and the corresponding optimization began node redeployment from the central region. Therefore, the position of the obstacle affects final deployment to some extent. The off-center obstacle caused worse node deployment, i.e., more twisted structures, as shown in the bottom panels of Figure 7.

**Figure 8:**
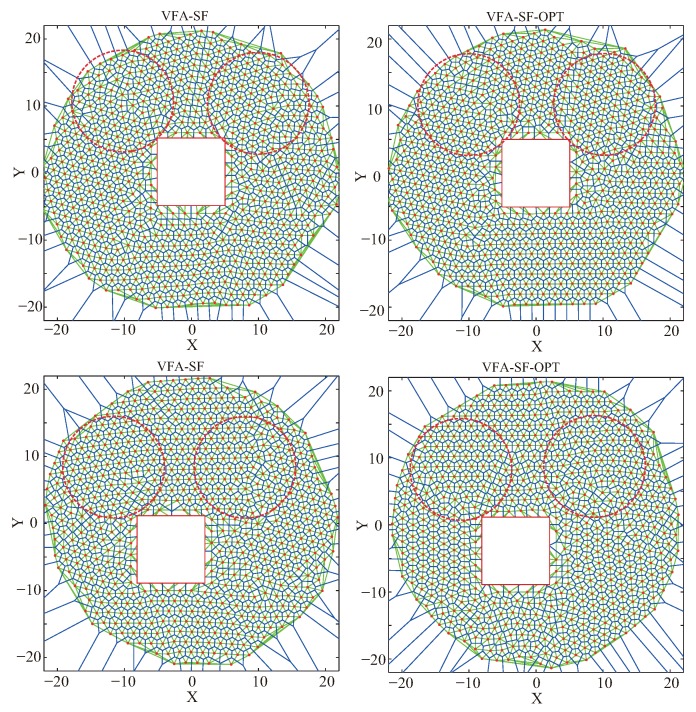
Final node deployments from (**left**) the VFA-SF and (**right**) the VFA-SF-OPT for the square obstacle cases: (**top**) when square obstacle is at the center; (**bottom**) when the square obstacle is off-center. Red dashed circles, region used to calculate the PCD values.

Similarly, Figure 8 shows the final node deployments for the square obstacle both at the center (top panels) and off-center (bottom panels) of the target region from the VFA-SF (left panels) and the VFA-SF-OPT (right panels). The square obstacle is also a central symmetry obstacle like a circle. Network deployment is significantly affected by four straight sides, however. This is because these straight sides break the normal balance of the hexagonal topology. By comparing with Figure 7, it is clear that the final deployments for the square obstacle have more twisted structures than the cases of the circle obstacle from both the VFA-SF and the VFA-SF-OPT. In such a case, the VFA-SF-OPT only optimizes the network topology in some extent but cannot obtain a perfect hexagonal network.

**Figure 9:**
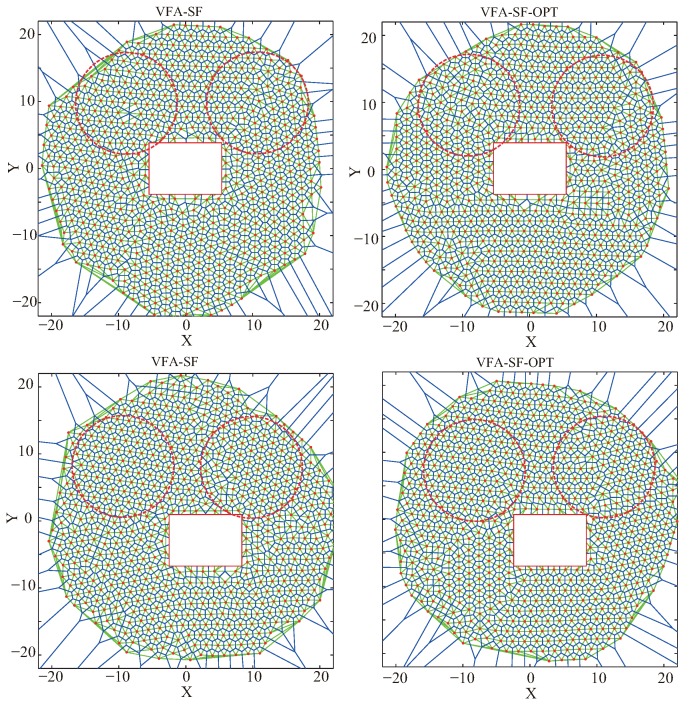
Final node deployments from (**left**) the VFA-SF and (**right**) the VFA-SF-OPT for the rectangular obstacle cases: (**top**) when rectangular obstacle is at the center; (**bottom**) when the rectangular obstacle is off-center. Red dashed circles, region used to calculate the PCD values.

We then considered a rectangular obstacle that had no central symmetry. Figure 9 presents the final node deployments for the rectangular obstacle, as shown in Figure 7 and Figure 8. It is obvious that the rectangular obstacle led to worse final node deployment than the square obstacle since noncentral-symmetry obstacles brought more edge effects to virtual-force calculations. In such a case, the VFA-SF-OPT cannot further improve network topology because the preferred central optimization by adding an external central force is invalid to some extent.

Moreover, Table 5 lists the corresponding computed PCD values for Figure 7, Figure 8 and Figure 9 from these obstacle tests. The regions for PCD calculations are indicated by the red dashed circles in the panels. It is clear that the VFA-SF-OPT usually obtained a smaller PCD value than the VFA-SF. Additionally, the final network from the circular obstacle was better than that from the square obstacle, while the final network from the square obstacle was better than the rectangular obstacle. It is obvious that the position, symmetry, and edge of the obstacle have great impact on the calculation of the virtual spring force and the external central force. In such a case, we the VFA-SF-OPT cannot be effectively applied to the irregular obstacle case.

## 5. Conclusions

A virtual-force algorithm inspired by spring force (VFA-SF) was introduced in which sensors moved by the spring force are determined from the relative position of neighboring nodes. Corresponding optimization by adding an external central force during force calculation was proved to be valid and effective. It helps to avoid coverage holes ans twisted structures, and increases the possibility of a final hexagonal topology in node deployment [13]. In this paper, based on the VFA-SF and the VFA-SF-OPT, the simulation results of different parameter effects during the deploying process were investigated in detail.

(1) As for the VFA-SF, statistical analysis indicates that, for maximum node velocity, a smaller $v_{max}$ causes worse node deployment in WSN applications. When $0.2<v_{max}<2.0$, the possibility of a perfect hexagonal network in the final deployment is stable at 42–50%. A larger $v_{max}$ helps to improve the final network deployment but does not increase the possibility of perfect hexagonal topology. Most importantly, it should be noted that the whole algorithm converges quicker as the $v_{max}$ limit increases.
(2) In the VFA-SF-OPT optimization, it is very clear that it is harder for smaller external virtual force to eliminate a coverage hole or twisted structure in the final node distribution. When external central force increases, it causes the compression of the central area during the process of optimization deployment. The nodes newly involved in the VFA-SF-OPT need to move for a longer distance and consume more energy. Therefore, experimental $F_{extern}$ should be slightly greater than the optimal value, which is determined based on the requirements of environment scenarios or location accuracy for some real WSN applications.
(3) Experiments of three typical obstacles were simulated by using both the VFA-SF and the VFA-SF-OPT. For the circle obstacle, two algorithms could still obtain good final distributions, and the VFA-SF-OPT had a better effect. However, for the square and rectangular obstacles, final node deployment was worse. This means that the VFA-SF and the VFA-SF-OPT are more useful in a central-symmetry obstacle event based on virtual-spring-force theory.

In summary, parameters have positive or negative effects on the performance and accuracy of the proposed VFA-SF and VFA-SF-OPT algorithms. In order to effectively achieve better hexagonal network topology and have better coverage performance for the target area, suitable parameters have to be selected in accordance with the experiment environments and application requirements, while a high overall coverage ratio is a goal.

## Figures and Tables

**Table 1:** Initial parameters used in simulation experiments of following subsections. Note: PCD, pair correlation diversion.

Category	Parameter Values
Basic parameters	N=500, m=1, $R_{s}=1$, $R_{c}=3$, $D_{m}=\sqrt{3}$
	$F_{centri}=0.05$, dt=0.08
Spring coefficient	κ=15
Damping coefficient	$\gamma_{critical}=\sqrt{4\kappa m}=7.75$
	$\gamma =1.08\times \gamma_{critical}$ (0–300 time steps)
	$\gamma =\gamma_{critical}$ ( after 300 time steps)
External central force	$F_{extern}=2.873$, the theoretical value in the reference [13]
	$F_{extern}=2,3.5,5.5$ in Figure 6
Radius to calculate PCD	$r_{T}=$18, 15, 10, respectively in Figure 1 of Section 4.1
	$r_{T}=$18 used in Section 4.2
	$r_{T}=$10 used in Section 4.3
	red dashed circles in Section 4.4

**Table 2:** Number of statistical cases of the PCD value evaluated from the final node deployment by the VFA-SF for different values of $v_{max}$.

PCD Value	$v_{\max}$ = 0.05	$v_{\max}$ = 0.1	$v_{\max}$ = 0.2	$v_{\max}$ = 0.6	$v_{\max}$ = 1.0	$v_{\max}$ = 2.0
0–0.05	5	6	23	19	24	22
0.05–0.10	0	0	2	2	0	0
0.10–0.15	3	4	4	2	3	2
0.15–0.20	4	1	4	4	2	2
0.20–0.25	3	7	5	3	3	9
0.25–0.30	6	7	4	5	5	3
0.30–0.35	4	9	4	5	4	4
0.35–0.40	9	9	1	4	7	5
0.40–0.45	12	4	3	5	2	1
>0.45	4	3	0	1	0	2

**Table 3:** Average equilibrium distance and average moving distance within radius = 10 for different values of external central force from the VFA-SF-OPT.

External Central Force	Average Node Distance	Average Moving Distance
0 (only spring force)	1.7317	1.2182
1	1.6964	1.6711
2	1.6805	2.1062
3.5	1.6682	2.6069
5.5	1.6642	2.7995
8	1.6602	3.0432
10	1.6563	3.0828

**Table 4:** Average equilibrium distance and average moving distance within radius = 10 for different values of external central force from the VFA-SF-OPT, when $v_{max}=0.3$, and 1.0, respectively.

$v_{\max}$	$F_{\mathrm{extern}}$	Node Distance	Moving Distance	$v_{\max}$	$F_{\mathrm{extern}}$	Node Distance	Moving Distance
0.3	0	1.7315	1.1759	1.0	0	1.7310	1.1759
	1	1.6942	1.6919		1	1.695	2.0404
	2	1.6819	2.7282		2	1.6796	2.6884
	3.5	1.6718	3.9669		3.5	1.6641	3.1902
	5.5	1.6692	4.2612		5.5	1.6587	3.4111
	8	1.6561	4.1812		8	1.6544	3.4891
	10	1.6557	4.1766		10	1.6529	3.4779

**Table 5:** PCD values calculated from red dashed circle in Figure 7, Figure 8 and Figure 9 for three typical obstacle cases.

Obstacle	Obstacle Position	PCD Values from the VFA-SF		PCD Values from the VFA-SF-OPT	
		Left Circle	Right Circle	Left Circle	Right Circle
circle	at center	0.1749	0.2564	0.0652	0.0680
circle	off-center	0.1866	0.1507	0.0865	0.0908
square	at center	0.3687	0.2979	0.1916	0.1569
square	off-center	0.3772	0.3549	0.2108	0.2915
rectangular	at center	0.4167	0.4038	0.2218	0.2556
rectangular	off-center	0.3824	0.5683	0.2040	0.2829
